# A highly sensitive immunosensor based on nanochannel-confined nano-gold enhanced electrochemiluminescence for procalcitonin detection

**DOI:** 10.3389/fchem.2023.1274424

**Published:** 2023-10-09

**Authors:** Qiang Chang, Xinhui Gu, Liming He, Fengna Xi

**Affiliations:** ^1^ Shanxi Bethune Hospital, Shanxi Academy of Medical Sciences, Taiyuan, China; ^2^ Tongji Hospital, Tongji Medical College, Huazhong University of Science and Technology, Wuhan, China; ^3^ Department of Chemistry, Zhejiang Sci-Tech University, Hangzhou, China

**Keywords:** electrochemiluminescence immunosensor, procalcitonin, nanochannel- confinement, signal amplification, gating effect

## Abstract

Sensitive detection of procalcitonin (PCT) in serum is crucial for the timely diagnosis and treatment of rheumatoid arthritis. In this work, an electrochemiluminescence (ECL) detection platform is developed based on *in-situ* growth of Au nanoparticles (AuNPs) in nanochannels and an analyte-gated detection signal, which can realize ECL determination of PCT with high sensitivity. Vertically ordered mesoporous silica films with amine groups and uniform nanochannel array (NH_2_-VMSF) is easily grown on the supporting indium tin oxide (ITO) electrode through electrochemical assisted self-assembly method (EASA). Anchored by the amino groups, AuNPs were grown *in-situ* within the nanochannels to catalyze the generation of reactive oxygen species (ROS) and amplify the ECL signal of luminol. An immuno-recognitive interface is constructed on the outer surface of NH_2_-VMSF, through covalent immobilization of PCT antibodies. In the presence of PCT, the immunocomplex will hinder the diffusion of luminol and co-reactants, leading to a gating effect and decreased ECL signals. Based on this principle, the immunosensor can detect PCT in the range from 10 pg/mL to 100 ng mL^-1^ with a limit of detection (LOD) of 7 pg mL^-1^. The constructed immunosensor can also be used for detecting PCT in serum. The constructed sensor has advantages of simple fabrication and sensitive detection, demonstrating great potential in real sample analysis.

## 1 Introduction

Procalcitonin (PCT) is a precursor protein produced by thyroid C cells. In normal conditions, PCT is present in the bloodstream at very low levels. However, when the body encounters severe infection or inflammatory response, the levels of PCT significantly increase (Tsujimoto et al., 2016; Sato et al., 2020). Rheumatoid arthritis is a chronic, autoimmune inflammatory disease. In recent years, PCT serves as a clinical biomarker and has value in terms of its role in inflammation mediation and prognosis evaluation in rheumatoid arthritis (Martinot et al., 2005; Zheng et al., 2015; Kato et al., 2023; Maleitzke et al., 2023). Therefore, it is crucial to detect PCT in serum with high sensitivity for timely diagnosis and treatment of rheumatoid arthritis (Butbul-Aviel et al., 2005; Akdoğan et al., 2021). Currently, several methods are available for determining PCT, including fluorescence immunoassay (Liu et al., 2021), surface plasmon resonance (Selimoğlu and Ayhan, 2023), chemiluminescence (Wang et al., 2020), etc. Among these methods, electrochemiluminescence (ECL) stands out as an analytical technique that combines the controllability of electrochemistry with the high sensitivity of chemiluminescence (Zheng et al., 2021). ECL offers advantages such as rapid detection, simple equipment setup, and low sample consumption (Sobhanie et al., 2022). Since protein biomarkers like PCT typically do not exhibit inherent ECL activity, the detection is commonly based on the altered signal of an ECL emitter when the biomarker binds to the recognition probe (Chen et al., 2022; Ma et al., 2022b; Huang et al., 2023). One widely used ECL emitter system is the luminol/H_2_O_2_ system, primarily due to its low excitation potential and cost-effectiveness (Wu et al., 2023). In this ECL system, H_2_O_2_ decomposes to generate reactive oxygen species (ROS), which then react with the electrochemically oxidized products of luminol (luminol^−•^) to produce excited 3-aminophthalic acid (3-AP^2-*^). The transition of the excited state back to the ground state generates ECL signals (Dong et al., 2023; Zhao et al., 2023). However, without a catalyst, the conversion rate of H_2_O_2_ to ROS may be slow, resulting in limited reaction kinetics and ECL intensity. Therefore, the development of an effective catalyst to accelerate H_2_O_2_ decomposition and enhance ECL signals holds significant importance for the sensitive detection of PCT.

Recently, there has been widespread attention to the development of highly sensitive ECL sensors by utilizing nanomaterials to catalyze the generation of ROS from H_2_O_2_ (Ding et al., 2020). Among these nanomaterials, gold nanoparticles (AuNPs) have been extensively employed in sensor construction due to their unique characteristics, including large specific surface area, high conductivity, and excellent catalytic performance (Huang et al., 2018; Hu et al., 2019; Liu et al., 2019; Fang et al., 2021). It has been demonstrated that AuNPs can effectively catalyze the generation of •OH and O_2_^•-^ from H_2_O_2_ (Hu et al., 2019). However, pure AuNPs without protective ligands are susceptible to instability under certain conditions. For instance, physiological saline (0.9% NaCl) can disrupt the double layer on the surface of AuNPs, leading to their aggregation and degradation. Additionally, the non-specific adsorption of proteins present in complex samples can obstruct the active sites of AuNPs, thereby influencing their catalytic activity (Sobhanie et al., 2022). Therefore, it is crucial to synthesize stable AuNPs to ensure their catalytic activity in the analysis of complex samples such as serum.

Porous materials possess a high specific surface area and adjustable pore size, making them ideal matrices for integrating functional nanomaterials (Gong et al., 2022a; Zhou et al., 2022; Zhao et al., 2023b; Deng et al., 2023; Zhu et al., 2023). Previous studies have demonstrated that confining AuNPs or graphene quantum dots within porous materials effectively enhances their stability and catalytic activity (Ding et al., 2014; Zhang et al., 2023a; Xu et al., 2023). Recently, there has been growing interest in modifying electrodes with vertically ordered mesoporous silica film (VMSF), which can create a nanochannel array on the supporting electrode (Han et al., 2022; Cui et al., 2023; Zhang et al., 2023c). With its ultrathin nanofilm structure (adjustable thickness of 20–200 nm), VMSF features perpendicular nanochannels to the substrate electrode, exhibiting a high density (∼40,000 nanochannels/μm^2^) and uniform size (commonly with diameters of 2–3 nm) (Liu et al., 2020; Yan et al., 2021; Yang et al., 2022). This unique structure provides VMSF-modified electrodes with distinct advantages. Firstly, the nanoscale thickness and ultra-high nanochannel density of VMSF enable effective diffusion and mass transfer, ensuring excellent permeability for small molecule analytes such as ECL probes or co-reactants (Luo et al., 2022; Zhang et al., 2022; Yan et al., 2023). Secondly, the VMSF electrode possesses a size exclusion effect due to the screening ability of its ultrasmall nanochannels, allowing only small molecules to pass through while effectively blocking biological macromolecules (e.g., proteins) and solid particles (Ma et al., 2020; Zheng et al., 2022a; Zheng et al., 2022b; Huang et al., 2022; Zou et al., 2022). Consequently, contamination from proteins and solid particles on the substrate electrode can be eliminated. This feature is particularly beneficial for constructing a signal-gated detection system. For instance, when the recognition ligand is fixed on the outer surface of VMSF, i.e., at the entrance of the nanochannel, the recognition and binding of the large-sized biomarkers will affect the diffusion of ECL probes/co-reactants towards the electrode surface, leading to gated ECL signal (Zhang et al., 2023b; Zhou et al., 2023). Additionally, VMSF channels can be utilized for *in-situ* growth of nanoparticles (Ding et al., 2014). The nanocavity ensures the small size of the nanoparticles while maintaining their stability without the need for protective ligands. Consequently, VMSF-based electrodes possess significant potential in fabricating ECL sensors for highly sensitive detection of PCT.

In this work, a signal-gated ECL immunosensor is fabricated based on *in-situ* growth of AuNPs in VMSF nanochannels and covalent immobilization of PCT antibody (Ab) on the outer surface of VMSF, which can realize sensitive detection of PCT with high sensitivity. When amino groups modified on the surface of VMSF nanochannels are used as anchor points, AuNPs are grown *in situ* to catalyze the decomposition of H_2_O_2_ to produce ROS, which significantly enhanced the ECL signal of luminol/H_2_O_2_ system. When PCT binds to the immuno-recognitive interface, the ECL signal reduces due to the hindered diffusion of small molecules to the supporting electrodes. Based on this mechanism, the constructed immunosensor can achieve ECL detection of PCT with high sensitivity.

## 2 Materials and methods

### 2.1 Chemicals and materials

PCT antigen and antibody were purchased from Beijing KEY-BIO Biotech Co., Ltd. (Beijing, China). Tetraethoxysilane (TEOS), cetyltrimethylammonium bromide (CTAB), potassium ferricyanide (K_3_[Fe(CN)_6_]), luminol, hydrogen peroxide (H_2_O_2_) and sodium hydroxide (NaOH) were purchased from Shanghai Aladin Biochemical Technology Co., LTD. (Shanghai, China). Phosphate buffer solution (PBS, 0.01 M, pH 7.4) was prepare using NaH_2_PO_4_ and Na_2_HPO_4_. Human serum (healthy person) was provided by Shanxi Bethune Hospital for real sample analysis. Indium tin oxide (ITO) conductive electrode (square resistance < 17Ω/sq, ITO thickness: 100 ± 20 nm) was purchased from Zhuhai Kaiwei Optoelectronics Technology Co., LTD. (Shenzhen, China). Before use, ITO was first washed with NaOH solution (1 M) before use, and then sonicated in acetone, ethanol, and deionized water for 30 min, respectively. All other chemicals were of analytical grade. Ultra-pure water (18.2 MΩ cm) was used in the whole experiment.

### 2.2 Measurements and instrumentations

The morphology of NH_2_-VMSF was characterized by transmission electron microscopy (TEM, HT7700, Hitachi, Tokyo, Japan) at an accelerating voltage of 100 kV. To prepare the sample for TEM characterization, NH_2_-VMSF was mechanically stripped from the surface of ITO electrode. Then, it was dispersed in ethanol, and finally dropped onto the copper mesh. Electrochemical impedance spectroscopy (EIS) and cyclic voltammetry (CV) measurement were all performed on an Autolab (PGSTAT302 N) electrochemical workstation (Metrohm, Switzerland). The ECL test was carried out on MPI-E II (Xi ‘an Ruimai Analytical Instruments Co., LTD., Xian, China). X-ray photoelectron spectroscopy (XPS) analysis was performed on a PHI5300 electron spectrometer (PE Ltd., USA) using Mg Kα radiation (250 W, 14 kV). The photomultiplier voltage was set to 600 V. Conventional three-electrode systems were used for electrochemical and ECL measurements. Briefly, a bare or modified ITO electrode was used as the working electrode. A platinum sheet (1 cm × 1 cm) was used as the counter electrode and an Ag/AgCl electrode (saturated using KCl) was the reference electrode.

### 2.3 Construction of immunosensors

Firstly, amino-functionalized VMSF (NH_2_-VMSF) was grown on the supporting ITO electrode by electrochemically assisted self-assembly (EASA) method (Ma et al., 2022a; Gong et al., 2022b; Gong et al., 2022c). To prepare the precursor solution for VMSF growth, ethanol (20 mL) and NaNO_3_ (20 mL, 0.1 M) was mixed. Afterwards, TEOS (2.732 mL), APTES (0.318 mL), and CTAB (4.35 mM) were added under stirring. The as-prepared mixture was stirred for 2.5 h after the pH was adjusted to 3 with HCl (6 M). Then, ITO was immersed in the precursor solution and a constant current of −350 μA was applied for 10 s, followed by thorough cleaning with ultrapure water. The resulting electrodes were dried under N_2_ and aged overnight at 120°C. The CTAB surfactant micelles (SM) are still present in the amino-functionalized VMSF channels of the obtained electrode (SM@NH_2_-VMSF/ITO). SM can be removed by stirring the electrode in 0.1 M HCl/ethanol solution for 5 min to obtain open nanochannels modified electrode (NH_2_-VMSF/ITO).

AuNPs were then *in-situ* synthesized in the nanochannels using the published method (Wu et al., 2015). Briefly, NH_2_-VMSF/ITO electrode was soaked in HAuCl_4_ solution (0.5%), applying a constant voltage of −0.5 V for 2 s. Then, the electrode (AuNPs@NH_2_-VMSF/ITO) was flushed with ultrapure water.

PCT antibody (Ab) was covalently immobilized on the outer surface of AuNPs@NH_2_-VMSF/ITO electrode using glutaraldehyde (GA) as bi-functional linker. Briefly, AuNPs@NH_2_-VMSF/ITO electrode was immersed in GA solution (0.5%) for 30 min at 37°C. After thoroughly rinsed with ultrapure water, the obtained electrode was immersed in Ab solution (10 μg/mL) to covalently immobilize Ab through Schiff base reaction between amino groups and aldehyde groups (Ab/AuNPs@NH_2_-VMSF/ITO). To block non-specific sites, the electrodes were incubated in BSA solution (1%, w/w) for 60 min obtain the immunosensor, BSA/Ab/AuNPs@NH_2_-VMSF/ITO.

### 2.4 Detection of PCT based on gated ECL signal

To detect PCT, the immunosensor Ab/GA/AuNPs@NH_2_-VMSF/ITO electrode was incubated with different concentrations of PCT at 37°C for 60 min. The ECL signals before and after binding of PCT were then measured in PBS (0.01 M, pH = 7.4) containing luminol (50 μM) and H_2_O_2_ (50 μM). For real sample analysis, human serum was diluted by a factor of 50 using PBS (0.01 M, pH = 7.4). PCT was detected using standard addition method. Specifically, after adding a certain amount of PCT artificially into the serum, serum samples are diluted for testing.

## 3 Results and discussion

### 3.1 Fabrication of immuosensor for ECL determination of PCT based on nanochannel-confined AuNPs and immuno-recognitive interface on the outer surface of VMSF

**FIGURE 1:**
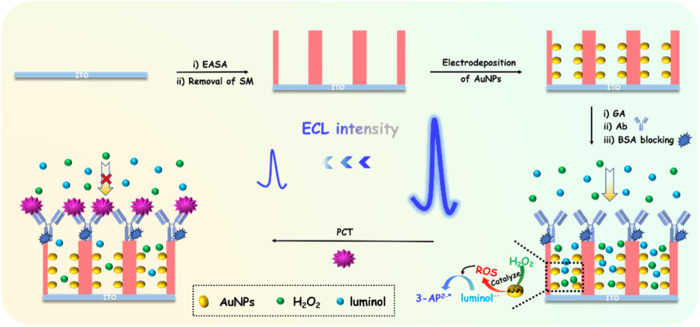
Schematic illustration for the construction of immunosensor based on nanochannel-confined AuNPs and immuno-recognitive interface on the outer surface of VMSF for ECL determination of PCT.

VMSF has a rigid structure, and its nanochannel array and outer surface can be functionalized as two independent regions. Figure 1 is the schematic illustration for the construction of immunosensor based on nanochannel-confined AuNPs and immuno-recognitive interface on the outer surface of VMSF for ECL determination of PCT. Using siloxanes containing amino group as precursor, VMSF with amino groups (NH_2_-VMSF) could be grown on the surface of ITO electrode through the EASA method (NH_2_-VMSF/ITO). On the one hand, amino groups can serve as anchor sites for *in-situ* growth of AuNPs. As shown, AuNPs are *in-situ* grown in the confined space of nanochannels through electro-deposition method (AuNPs@NH_2_-VMSF/ITO), showing advantages of controllable particle size, no need for additional stabilizers and high catalytic performance. The as-synthesized AuNPs can act as catalysts and promote the generation of ROS from H_2_O_2_, which then reacts with luminol^−^• to generate 3-AP^2-^*. The increase in ROS concentration will significantly increase the generated ECL signals. On the other hand, amino groups can be used as reactive sites to covalently immobilize the recognition antibody (Ab) of PCT after derivatization with the bifunctional agent, glutaraldehyde (GA). Thus, an immuno-recognitive interface is fabricated on the outer surface of VMSF(BSA/Ab/AuNPs@NH_2_-VMSF/ITO). When PCT exists, non-conductive immunocomplexes are formed due to the specific binding between antigen and antibody, which reduces the diffusion and mass transfer of ECL probe or co-reactants to the electrode surface and reduces ECL signals. Based on this gated ECL signal, highly sensitive ECL detection of PCT can be achieved. Due to its silica structure, VMSF will not swell during use and has high binding stability with ITO. This overcomes the disadvantage of low stability of commonly used polymer film modified electrodes, which are prone to swelling during use. Thus, the fabricated immunosensor based on the *in-situ* growth of AuNP catalyst in nanochannels and the biological recognition interface on the outer surface of VMSF has the advantages of simple preparation and stable modification layer.

### 3.2 Characterization of NH_2_-VMSF and AuNPs@NH_2_-VMSF modified electrode

**FIGURE 2:**
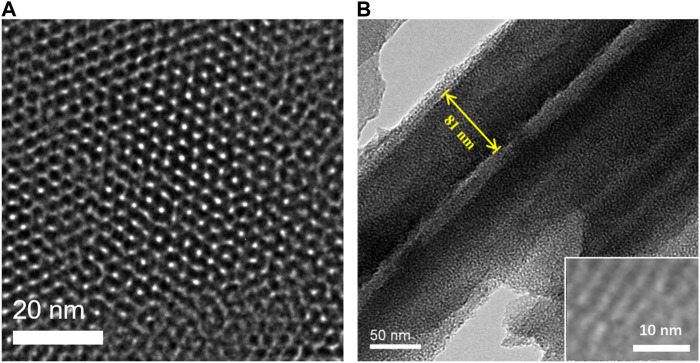
**(A)** Top-view and **(B)** cross-sectional TEM images of NH_2_-VMSF. Inset in B is the HRTEM image.

The morphology of NH_2_-VMSF is characterized by transmission electron microscopy (TEM). The top-view and cross-sectional TEM of NH_2_-VMSF/ITO are shown in Figure 2, respectively. From the top-view TEM image, a porous structure of NH_2_-VMSF with no defect is revealed. The pores are arranged in a hexagonal structure with an approximate pore size of 2.6 nm (Figure 2A). Measured with ImageJ software, the pore density is 7.8 × 10^12^/cm^2^, corresponding to a porosity of 42%. From the cross-sectional TEM view (Figure 2B), nanochannel file with thickness is about 90 nm is observed.

**FIGURE 3:**
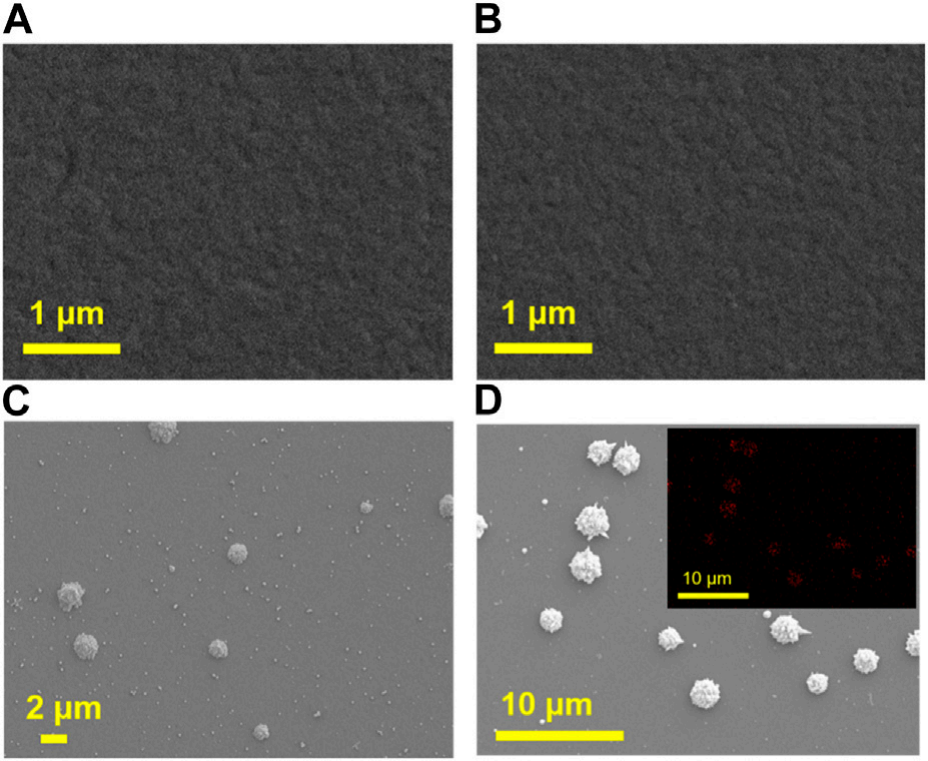
SEM images of NH_2_-VMSF/ITO before **(A)** and after electrodeposition of AuNPs for 2 s **(B)**, 5 s **(C)** or 10 s **(D)**. Inset in d is the Au element mapping image.

Subsequently, the deposition of AuNPs is proceeded. If AuNPs, especially large-sized ones, were deposited on the outer surface of VMSF, they would block the nanochannels, hindering the diffusion of small electrochemical probes to reach the surface of the underlying electrode. Therefore, it is necessary to deposit AuNPs inside the nanochannels to ensure their small size without significantly affecting diffusion. Thus, it is necessary to deposit AuNPs inside the nanochannels to ensure their small size, without significantly affecting diffusion. To verify that AuNPs could be *in-situ* localized to nanochannels of NH_2_-VMSF/ITO, scanning electron microscope (SEM) characterization is performed. Figure 3 shows the SEM images of NH_2_-VMSF/ITO before and after AuNPs electrodeposition for different time. As seen, no significant change is observed on the surface of NH_2_-VMSF/ITO before and after 2 s electrodeposition of AuNPs (Figures 3A, B). However, many small spheres with micrometer size and shape with burr appear on the outer surface of NH_2_-VMSF when the deposition time extends to 5 s or 10 s (Figures 3C, D). The element mapping image presented in the inset of Figure 3D confirms that the presence of Au nanomaterials. Thus, AuNPs are deposited in the nanochannels when the deposition time is short. As the deposition time increases, large gold nanomaterials will be formed on the outer surface of VMSF and the particle size increases with longer deposition time. In further experiments, electrode with deposited AuNPs in nanochannels is investigated and recorded as AuNPs@NH_2_-VMSF /ITO.

**FIGURE 4:**
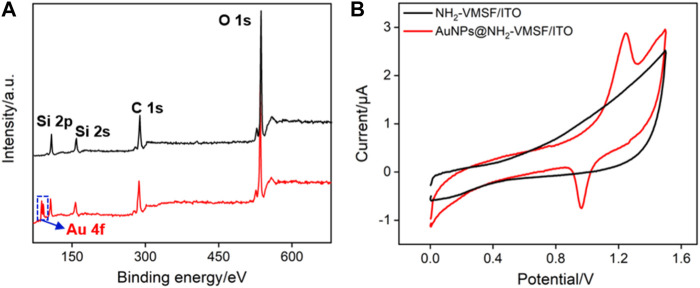
**(A)** XPS survey spectrum obtained on NH_2_-VMSF/ITO or AuNPs@NH_2_-VMSF/ITO electrode. **(B)** CV curves obtained on NH_2_-VMSF/ITO and AuNPs@NH_2_-VMSF/ITO electrodes in 0.5 M H_2_SO_4._

The changes in elemental composition before and after the deposition of AuNPs on NH_2_-VMSF/ITO electrodes were investigated using X-ray photoelectron spectroscopy (XPS). As shown in Figure 4, characteristic peak of Au element is observed on AuNPs@NH_2_-VMSF/ITO electrode. The existence of AuNPs on the nanochannels is further verified through cyclic voltammetry (CV) characterization. Compared with NH_2_-VMSF/ITO electrode, sharp oxidation and reduction peaks corresponding to the redox reaction of AuNPs on the electrode surface are observed, indicating the successful electrodeposition of AuNPs in nanochannels of NH_2_-VMSF.

### 3.3 Feasibility for the construction of immunosensors

**FIGURE 5:**
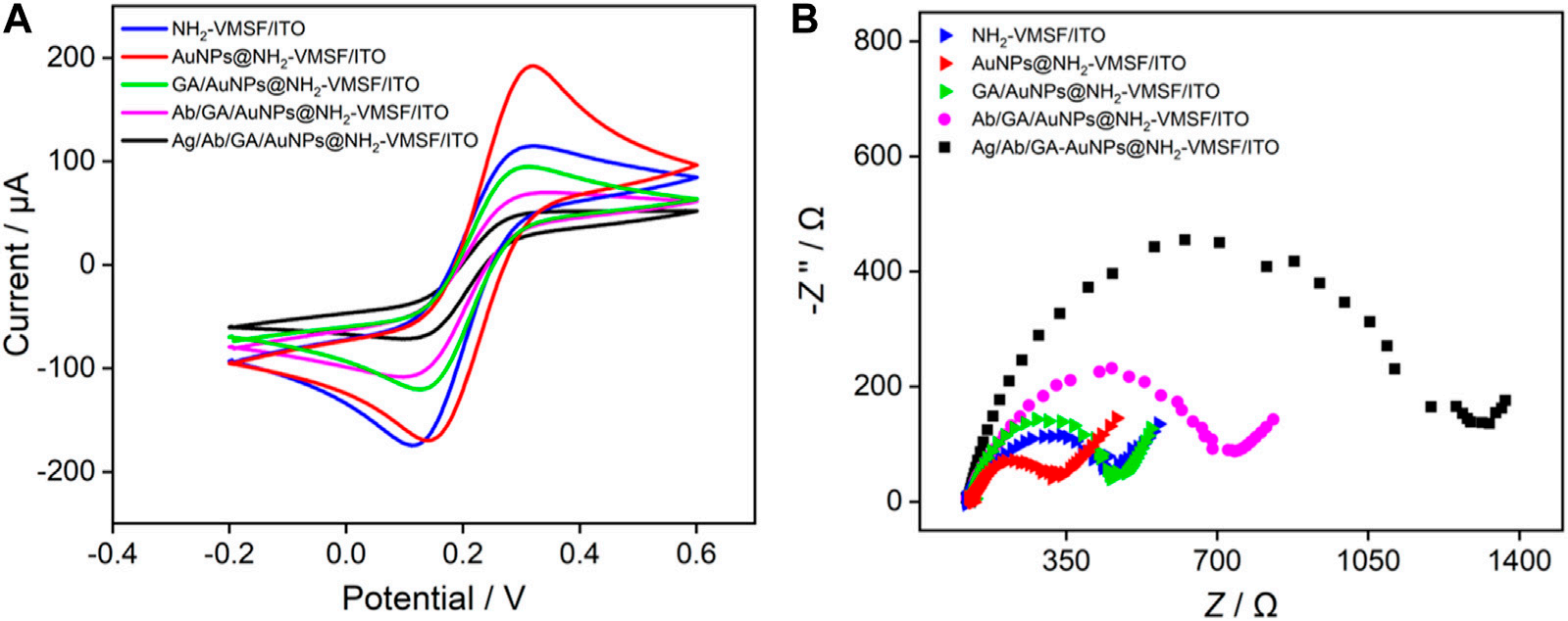
CV **(A)** and EIS **(B)** plots obtained on different electrodes in 0.1 M KCl solution containing 2.5 mM Fe(CN)_6_^3-/4-^. The concentration of PCT was 80 pg/mL.

Using Fe(CN)_6_^3-/4-^ as the standard electroactive probe, the changes in the electrode interface during the construction of the immunosensor are investigated using CV and electrochemical impedance spectroscopy (EIS). The results are shown in Figure 5. Compared with NH_2_-VMSF/ITO, the redox peak current of Fe(CN)_6_^3-/4-^ significantly increases after electrodeposition of AuNPs (Figure 5A). In addition, the peak to peak difference also remarkably decreased owing to good conductivity of AuNPs. After covalent immobilization of Ab, as well as blocking non-specific sites using bovine serum albumin (BSA), the redox peak current of Fe(CN)_6_^3-/4-^ decreases sequentially, indicating further reduce of electron transfer. This is due to the fact that the protein structure of immobilized antibody or BSA for non-specific blocking is not conductive, which increases the interface resistance on the electrode surface. The EIS curves also confirm the same conclusion (Figure 5B). As shown, the charge transfer resistance (*R*ct) gradually changes as the electrode is gradually modified. Briefly, *R*ct for NH_2_-VMSF/ITO electrode is 323 Ω. After deposition of AuNPs, the *R*ct of the electrode decreases to 238 Ω because AuNPs with high conductivity can act as electronic wire. When Ab was covalently immobilized at the electrode interface followed with BSA blocking, *R*ct significantly increases to 567 Ω. In presence of PCT, the redox peak current of Fe(CN)_6_^3-/4-^ significantly decreases accompanying with the increase of *R*ct (942 Ω), confirming that the immune recognition interface can specifically recognize PCT. The above results demonstrate the successful construction of the immunosensor.

### 3.4 AuNPs sensitized ECL signal and gated signal caused by binding of PCT

**FIGURE 6:**
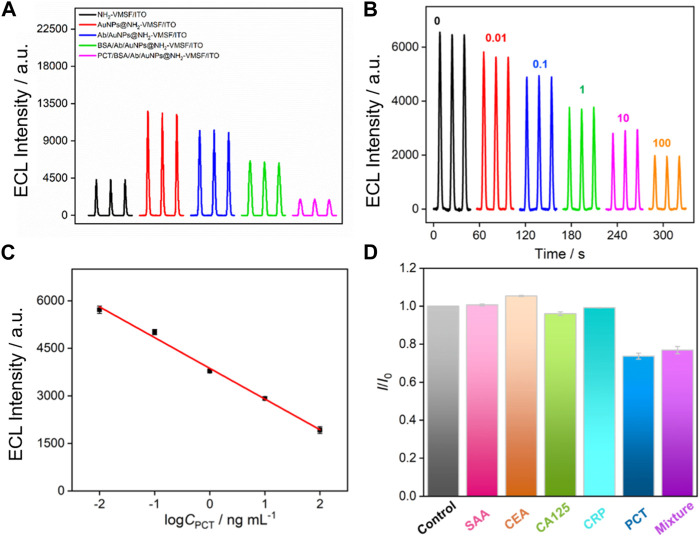
**(A)** ECL responses of different electrodes in PBS solution containing luminol and H_2_O_2_. **(B)** ECL responses of the fabricated immunosensor in presence of different concentrations of PCT. **(C)** The corresponding calibration curves. Error bars represent the standard deviation of three measurements. **(D)** Relative current ratio (*I*/*I*_0_) obtained on the developed immunosensors before (*I*_0_) and after (*I*) incubation with buffer (control), SAA, CEA, CA125, CRP, PCT, or their mixture. The concentrations of all the species are 10 pg/mL.

Figure 6 shows the ECL signals obtained on different electrodes in luminol/H_2_O_2_ system. Compared with NH_2_-VMSF/ITO electrode, AuNPs@NH_2_-VMSF/ITO electrode exhibits significantly increased ECL signal. In the presence of AuNPs, the ECL signal increases by a factor of 2.9. On the one hand, the excellent conductivity of AuNPs can facilitate the electron transfer on the electrode surface. On the other hand, AuNPs are catalysts that can catalyze H_2_O_2_ to generate rich ROS in nanochannels, which reacts with luminol^−•^ to generate more 3-AP2^-*^ and improves ECL signal. Thus, AuNPs within the nanochannel can enhance the ECL signal of the electrode, that is beneficial for improving the detection sensitivity of the fabricated sensor. To investigate the enhancement mechanism of AuNPs on the ECL of the luminol-H_2_O_2_ system, free radical scavengers were employed to examine the types of ROS during the ECL process. As shown in Supplementary Figure S1, upon the addition of the superoxide anion (O^2·-^) scavenger, benzoquinone (BQ), and the hydroxyl radical (·OH) scavenger, tert-butanol (TBA), the ECL signals significantly decreased. This suggests that the ROS involved in this ECL process are O^2·-^ and ·OH, with O^2·-^ being the predominant species. Specifically, ·OH is generated due to the catalytic action of AuNPs acting as a mimic peroxidase on H_2_O_2_, while O^2·-^ likely originates from AuNPs acting as electron-enhancing materials that promote the electrocatalytic oxidation of H_2_O_2_.

When the immuno-recognitive interface is employed to recognize PCT, the ECL signal of the electrode remarkably decreases, indicating the specific binding of PCT. Owing to the large size and non-conductive characteristics of the immunocomplex formed on the entrance of the nanochannel, the diffusion of luminol and H_2_O_2_ molecules towards the electrode surface is hindered, thereby reducing ECL signals. Therefore, the binding of PCT can generate a gating effect on the ECL signal of the electrode.

### 3.5 ECL determination of PCT using the constructed immunosensor

The performance of the constructed immunosensor for ECL determination of PCT is investigated. As shown in Figure 6B, ECL signal obtained on the immunosensor gradually decreased as the concentration of PCT increases, which is attributed to the signal gating effect of the formed immunocomplex after PCT binding. A good linear relationship is revealed between the ECL signal intensity (*I*_ECL_) and the logarithmic value of PCT concentration (log*C*) when the concentration of PCT ranges from 0.01 ng mL^-1^–100 ng mL^-1^ (Figure 6C). The linear equation is *I*_ECL_ = 3,870–969log*C* (*R* ^2^ = 0.992). The limit of detection (LOD) is 7 pg mL^-1^. Table 1 demonstrates comparison of PCT detection performance using different methods (Li et al., 2015; Ghrera, 2019; Molinero-Fernández et al., 2019; Gao et al., 2020; Xu et al., 2021a; Xu et al., 2021b; Yue et al., 2022). The LOD obtained on the fabricated sensor is lower than that obtained using ITO electrode modified with quantum dots (QD) labelled Ab (Ghrera, 2019), or sandwich-type electrochemical immunoassay based on magnetic beads (MBs) (Molinero-Fernández et al., 2019), or g-C_3_N_4_-NiCo_2_S_4_-carbon nanotubes-silver nanoparticles (g-C_3_N_4_-NiCo_2_S_4_-CNTs-AgNPs) sensor (Xu et al., 2021b). In addition, the sensor developed in this study only requires the integration of a nanochannel film on cheap ITO electrode followed by the simple electrodeposition of AuNPs within the nanochannel, making it a disposable, cost-effective sensor. Compared to other sensors, the fabrication of the developed sensor is simple and does not require extensive modification of nanomaterials.

**TABLE 1:** ECL determination of PCT in human serum.

Sample	Added^b^ (ng mL^-1^)	Found (ng mL^-1^)	Recovery (%)	RSD (%, n = 3)
serum	0.0100	0.00998	99.8	4.1
	0.100	0.101	101	2.1
	1.00	0.999	99.9	2.3

The selectivity of the fabricated immunosensors is investigated using several protein biomarkers. When serum amyloid A (SAA), carcinoembryonic antigen (CEA), cancer antigen 125 (CA125), or C-reactive protein (CRP) is incubated with the constructed immunosensor, no significant change in ECL signals is observed (Figure 6D). On the contrary, PCT or the mixture containing PCT and the above proteins can significantly reduce the ECL signal of the electrode, proving that the constructed immunosensor has good selectivity.

### 3.6 Real sample analysis

To verify the ability of the constructed sensor in practical applications, the detection performance of PCT is investigated using standard addition method with serum as the real sample (diluted by a factor of 50). As shown in Table 1, the recovery for PCT detection is within the range of 99.8%–101%, and the relative standard deviation (RSD) measured is less than 4.1%, confirming the accuracy of the developed immunosensor in analysis of real sample.

## 4 Conclusion

In summary, an ECL immunosensor is fabricated based on the catalytic amplification of ECL signals by nanochannel-confined AuNPs and the gating effect caused by the analyte, which can achieve highly sensitive detection of PCT. AuNPs generated *in situ* and localized within nanochannels catalyze the generation of ROS from H_2_O_2_, thereby significantly improving the ECL signal of luminol. In addition, the outer surface of nanochannels can be used to immobilize specific antibodies, effectively constructing immuno-recognitive interface. The immunocomplex formed after capture of PCT reduces the diffusion of ECL probe and co-reactant, generating gating effect and decreased signals. The constructed immunosensor has the advantages of simple fabrication and high sensitivity, demonstrating great potential in biological analysis, medical detection, etc.

## Data Availability

The original contributions presented in the study are included in the article/Supplementary Material, further inquiries can be directed to the corresponding author.
