# Supplementary material for: A highly sensitive immunosensor based on nanochannel-confined nano-gold enhanced electrochemiluminescence for procalcitonin detection
Source: Front Chem. 2023 Oct 9;11:1274424. doi: 10.3389/fchem.2023.1274424 (PMC10591179; doi:10.3389/fchem.2023.1274424)
Supplement: Supplementary file 1 [file DataSheet1.pdf]

Supporting Information

TABLE S1 | Comparison of the PCT detection performance using different methods.

**Electrode materials**	**Method**	**Detection range (ng/mL)**	**LOD (pg/mL)**	**Ref.**
GOx@Ab_2-PtNPs-Fc-C_{60}/PCT/BSA/Ab_1/ AuNPs@MWCNTs/GCE	EC	0.01-10	6	Li et al. 2015
BSA/QD-Ab/AS/ITO	EC	10-10000	210	Ghrera 2019
BSA/Ab/NiFe PBA nanocubes@TB/GCE	EC	0.001-25	0.3	Gao et al. 2020
HRP-Ab_2/PCT/Ab_1/MBs/SPCE	EC	0.25-100	90	Molinero-Fernández et al. 2019
NBA-UiO-67-Ab_2/PCT/Ab_1/BSA/g-C_3N_4-AgNPs/GCE	EC	0.005-50	1.67	Yue et al. 2022
BSA/Ab/g-C_3N_4-$NiCo_2S_4$-CNTs-AgNPs/GCE	EC	0.05-50	16.7	Xu et al. 2021b
Fca-ZIF-8-Ab_2/PCT/BSA/Ab_1/RuSiNPs-g-C_3N_4/ITO	ECL	0.005-100	0.85	Xu et al. 2021a
Ab/GA/AuNPs@NH_2-VMSF/ITO	ECL	0.01-100	7	This Work

GOx: Glucose oxidase; PtNPs: Platinum nanoparticles; Fc: Ferrocene carboxylic acid; C_{60}: Amino group functionalised C_{60} nanoparticles; BSA: Bovine serum albumin; MWCNTs: Multi-walled carbon nanotubes; GCE: Glass carbon electrode; QD: CdSeZnS quantum dots; AS: (3-eaminopropyl)trimethoxysilane; NiFe PBA nanocubes@TB: Toluidine blue functionalized NiFe Prussian-blue analog nanocubes; HRP: Horseradish peroxidase; MBs: Magnetic beads; NBA: Nile blue A; UiO-67: Zr-based metal organic framework; g-C_3N_4: Graphitic carbon nitride; AgNPs: Silver nanoparticles; $NiCo_2S_4$: Nickel cobalt sulfide; CNTs: Carbon nanotubes; Fca： Ferrocene carboxylic acid; ZIF-8: Zeolitic Zn^{2+}-imidazolate cross-linked frame-work; RuSiNPs: RuSi nanoparticles.

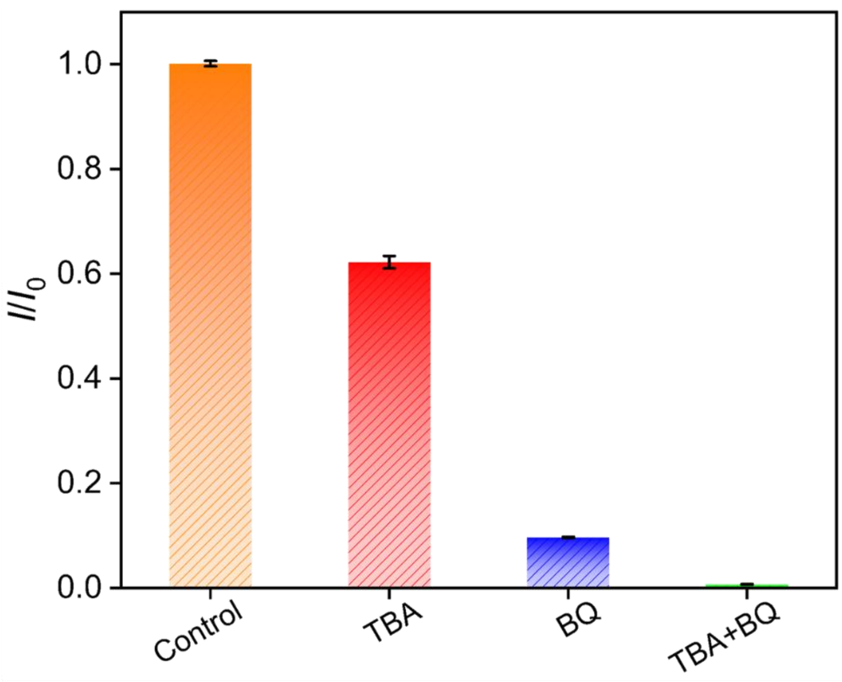

Figure S1 The ECL response obtained on AuNPs@NH_2-VMSF/ITO electrode in luminol-H_2O_2 system in absence or presence of different radical trapping agents in 0.01 M PBS (pH=7.4) buffer solution. *I* and I_0 are the ECL signals in the presence or absence of free radical capture agents, respectively. The concentrations of 1,4-benzoquinone (BQ) and tert-butanol (TBA) are 100 μM and 100 μg/mL, respectively.
